# Voclosporin for Lupus Nephritis: A #NephJC Editorial on AURORA

**DOI:** 10.1016/j.xkme.2021.10.001

**Published:** 2021-10-22

**Authors:** Bourne Auguste, Jade Teakell, Avinash Rao Ullur, Joel M. Topf, Swapnil Hiremath

**Affiliations:** 1 Department of Medicine, University of Toronto, Toronto, ON, Canada; 2 Department of Medicine, McGovern Medical School, University of Texas Health Science Center, Houston, TX; 3 Department of Medicine, Oakland University William Beaumont School of Medicine, Rochester, MI; 4 Department of Medicine, University of Ottawa, Ottawa, Ontario, Canada

**Keywords:** AURORA, lupus nephritis, #NephJC, Twitter journal club, voclosporin

*#NephJC is a recurring Twitter-based journal club. #NephJC editorials highlight the discussed article and summarize key points from the NephJC TweetChat.*

### Introduction

Kidney involvement in systemic lupus erythematosus (SLE) is common with 40% to 70% of patients who develop some form of lupus nephritis. Approximately 6% to 19% of patients with lupus nephritis will develop end-stage kidney disease within a decade of an SLE diagnosis.^1^^,^^2^ Patients with lupus nephritis are usually treated with immunosuppressive therapy that may include a combination of glucocorticoids, mycophenolate mofetil (MMF), or cyclophosphamide. There have been several controlled clinical trials in lupus nephritis, with both biologic agents and conventional cytotoxic and antimetabolite medications.^3^, ^4^, ^5^, ^6^, ^7^, ^8^, ^9^, ^10^ However, no drug has received formal US Food and Drug Administration approval for the treatment of lupus nephritis in the last decade. These agents continue to be used off-label, and have far-from-perfect response rates and considerable toxicities. For example, reduction in proteinuria within the first 6-12 months of initial treatment is recognized as an important prognostic marker for predicting disease flares, progression to end-stage kidney disease, and death.^11^^,^^12^ However, up to 60% of patients with lupus nephritis are unable to achieve guideline targets for proteinuria (urine protein-creatinine ratio (UPCR) < 0.5-0.7 mg/mg) within the first year of treatment with current therapeutic options.^13^^,^^14^ Belimumab, a recombinant human antibody that inhibits B-cell activating factor, was the first drug approved by the US Food and Drug Administration in 2011 for the treatment of SLE and has shown efficacy in lupus nephritis as well.^15^, ^16^, ^17^ Two trials have reported the efficacy of a calcineurin inhibitor (CNI), tacrolimus, which reduced proteinuria in patients with lupus nephritis.^18^^,^^19^ In light of this, voclosporin, a novel CNI initially developed for transplant recipients, has more recently pivoted for the management of lupus nephritis. Voclosporin is structurally similar to cyclosporine, except for one amino acid, and has a higher affinity for calcineurin binding along with more predictable pharmacokinetics.^20^ The phase 2 trial with voclosporin was AURA-LV (Aurinia Urinary Protein Reduction Active−Lupus With Voclosporin) which compared 2 voclosporin doses (23.7 mg and 39.5 mg twice a day) to placebo, on a background of steroids and MMF, to assess the efficacy and adverse events. There was an increase in complete response at 6 months for both doses, but only reaching statistical significance for the 23.7-mg dose. Serious adverse events occurred more often in both voclosporin groups, and more deaths occurred in the low-dose group compared to the placebo and high-dose voclosporin groups.^21^ This led to the current phase 3 trial, AURORA-1 (Aurinia Renal Response in Active Lupus With Voclosporin).

### The Study

AURORA-1 was a phase 3 trial to verify the efficacy and safety of voclosporin (as an add-on to MMF and steroids) to treat lupus nephritis. It was a global, multicenter, double-blind, randomized, placebo-controlled trial with sites in 27 countries across North and South America, Africa, Asia, and Europe. Patients with SLE, a kidney biopsy showing lupus nephritis (class III, IV, V; alone or in combination), and evidence of active disease on the basis of proteinuria were included. Notable among the exclusion criteria was estimated glomerular filtration rate (eGFR) <45 mL/min/1.73 m^2^. All patients received intravenous methylprednisolone for 2 days followed by a rapid oral prednisone taper, going down to 2.5 mg/day by week 16. They also received MMF up to 1 g twice a day. The intervention arm received 23.7 mg of voclosporin twice a day for 52 weeks (ie, 3 pills of 7.9 mg twice a day), and the control group received a matching placebo. The primary end point was a complete response at week 52, defined by UPCR ≤0.5 mg/mg, eGFR ≥60 mL/min, no need for rescue medications, and no more than 10 mg of prednisone per day in weeks 44-52. The secondary end points, analyzed hierarchically, included time to UPCR ≤0.5 mg/mg, partial response (UPCR reduction of ≥50%) at 24 or 52 weeks, and complete response at 24 weeks. The trial was powered for a 14.4% higher absolute increase in complete response rate (from 20% in placebo to 34.4% in voclosporin; odds ratio [OR], 2.1). The trial was funded by Aurinia Pharmaceuticals, the makers of voclosporin, who also collected, analyzed, and interpreted the data.

The trial successfully recruited 357 participants with lupus nephritis, of whom ∼90% were women, including a mix of ethnicities, and the majority having normal eGFR and class IV lupus nephritis.

A complete response was seen in 41% of patients treated with voclosporin compared to 23% of patients in the placebo group (OR, 2.65; 95% confidence interval [CI], 1.64-4.27; *P* < 0.0001). A difference in complete response at 24 weeks was also observed with 32% versus 20% of patients in the voclosporin versus the placebo group, respectively (OR, 2.23; 95% CI, 1.34-3.72; *P* = 0.002). Lastly, the voclosporin group had a shorter time to UPCR ≤0.5 mg/mg at 169 days compared to 372 days in the control group (hazard ratio, 2.02; 95% CI, 1.51-2.70; *P* < 0.001). Given the well-preserved kidney function based on eligibility, it was not surprising to note that the mean eGFR was less than 5 mL/min/1.73 m^2^ different from baseline, with no difference between groups. The rate of adverse events (most commonly, infections) was the same in both groups, 91% and 89%, and deaths occurred in 1 patient in the voclosporin group (<1%) compared to 5 in the control group (3%). Interestingly, gastrointestinal and nervous system disorders were almost twice as common with voclosporin compared to placebo, in keeping with the CNI side effect profile.

### The Tweetchat

**Figure 1:**
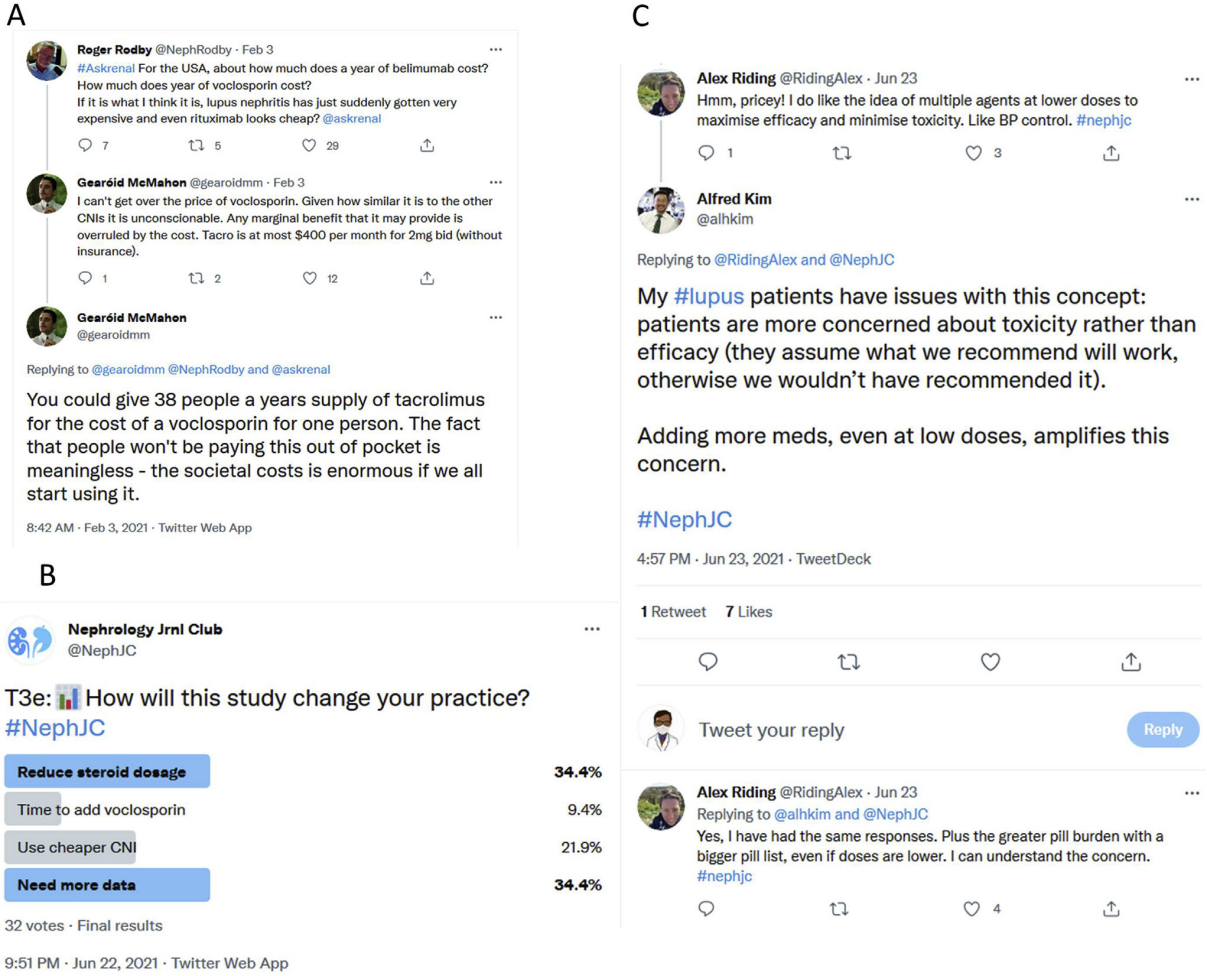
(A) A Twitter conversation covering the cost of voclosporin and it being quite high compared to existing calcineurin inhibitors. From https://twitter.com/gearoidmm/status/1356961327680430082?s=20. (B) A Twitter poll, where a plurality of respondents thought that they needed more data before using voclosporin, and that the major message of the trial was that lower doses of steroids could be used safely. From https://twitter.com/gearoidmm/status/1356961327680430082?s=20. (C) A Twitter discussion of the advantages of using multiple agents at low doses, with the opposite side of the coin being a greater patient concern of toxicity and pill burden. From https://twitter.com/alhkim/status/1407805140955013120?s=20.

The NephJC tweetchat discussion about AURORA-1 on June 22 and 23, 2021, included 162 nephrologists, patients, and rheumatologists. The participants tweeted 1,045 times. At baseline, less than 5% of the participants said that they would use CNIs as the first choice for induction in lupus nephritis, despite prior data on CNIs in lupus, and only reserved CNIs for class V or refractory situations. Most of the discussion centered around the use of proteinuria reduction as the outcome of choice, because the effect of CNIs on proteinuria is already known, and 2 previous trials have demonstrated the effect of CNIs (tacrolimus) on this outcome.^18^^,^^19^ Though the lack of nephrotoxicity was reassuring, the follow up of one year was thought to be short. It was also noted that the immunological parameters (eg, complement levels and antibodies) studied did not change significantly, though these are not reliable markers of disease activity in lupus nephritis. Some felt that a better comparator for voclosporin would have been another CNI (ie, cyclosporine or tacrolimus). The pricing plan, at $92,000 a year for voclosporin,^22^ produced severe sticker shock. Even with the advantage of avoiding therapeutic drug monitoring, this represents about 40 times the cost of tacrolimus, with no established advantage except for an approved indication (Fig 1A). On the bright side, there was an appreciation among the group members of the rapid taper regime used in the trial of steroid to 2.5 mg at week 16, as compared with the previous trials where the taper was usually slower, and the maintenance dose was 10 mg.^19^ This also came with a response rate in the placebo group of 20%, higher than usually seen in historical lupus nephritis trials.

Overall, less than 10% of the participants thought that they would use voclosporin in this setting, with a slightly higher preference to use another CNI and significant enthusiasm for the rapid steroid taper protocol (see Fig 1B for poll response). Voclosporin was a welcome addition to the therapeutic armamentarium but not considered to be a revolutionary addition.^23^ The discussants also had concerns about whether the increased number of medications, even at lower doses, incur more side effects (Fig 1C).
